# The impact of colistin-based regimens on mortality compared to other antimicrobials in patients with carbapenem-resistant Enterobacterales bacteremia in South African hospitals: a cross-sectional study

**DOI:** 10.1186/s12879-024-09459-x

**Published:** 2024-06-05

**Authors:** Nqobile Ngoma, Olga Perovic, Alex de Voux, Alfred Musekiwa, Liliwe Shuping, for GERMS-SA

**Affiliations:** 1 South African Field Epidemiology Training Programme, National Institute for Communicable Diseases a Division of National Health Laboratory Service, 1 Modderfontein Road, Sandringham, Johannesburg, 2131 South Africa; 2 Centre for Healthcare Associated-Infections, Antimicrobial Resistance and Mycoses, National Institute for Communicable Diseases a Division of National Health Laboratory Service, Johannesburg, South Africa; 3 School of Health Systems and Public Health, Faculty of Health Sciences, University of Pretoria, Pretoria, South Africa; 4 Department of Clinical Microbiology and Infectious Diseases, School of Pathology, Faculty of Health Sciences, University of the Witwatersrand, Johannesburg, South Africa; 5 Division of Epidemiology and Biostatistics, School of Public Health, University of Cape Town, Cape Town, South Africa

**Keywords:** Carbapenem-resistant Enterobacterales, Bacteremia, South Africa, Antimicrobial resistance, Colistin-based regimens, Healthcare-associated infections

## Abstract

**Background:**

Treatment of carbapenem-resistant Enterobacterales (CRE) infections in low-resource settings is challenging particularly due to limited treatment options. Colistin is the mainstay drug for treatment; however, nephrotoxicity and neurotoxicity make this drug less desirable. Thus, mortality may be higher among patients treated with alternative antimicrobials that are potentially less efficacious than colistin. We assessed mortality in patients with CRE bacteremia treated with colistin-based therapy compared to colistin-sparing therapy.

**Methods:**

We conducted a cross-sectional study using secondary data from a South African national laboratory-based CRE bacteremia surveillance system from January 2015 to December 2020. Patients hospitalized at surveillance sentinel sites with CRE isolated from blood cultures were included. Multivariable logistic regression modeling, with multiple imputations to account for missing data, was conducted to determine the association between in-hospital mortality and colistin-based therapy versus colistin-sparing therapy.

**Results:**

We included 1 607 case-patients with a median age of 29 years (interquartile range [IQR], 0–52 years) and 53% (857/1 607) male. *Klebsiella pneumoniae* caused most of the infections (82%, *n*=1 247), and the most common carbapenemase genes detected were *bla*_OXA-48-like_ (61%, *n*=551), and bla_NDM_ (37%, *n*=333). The overall in-hospital mortality was 31% (504/1 607). Patients treated with colistin-based combination therapy had a lower case fatality ratio (29% [152/521]) compared to those treated with colistin-sparing therapy 32% [352/1 086]) (*p*=0.18). In our imputed model, compared to colistin-sparing therapy, colistin-based therapy was associated with similar odds of mortality (adjusted odds ratio [aOR] 1.02; 95% confidence interval [CI] 0.78-1.33, *p*=0.873).

**Conclusion:**

In our resource-limited setting, the mortality risk in patients treated with colistin-based therapy was comparable to that of patients treated with colistin-sparing therapy. Given the challenges with colistin treatment and the increasing resistance to alternative agents, further investigations into the benefit of newer antimicrobials for managing CRE infections are needed.

**Supplementary Information:**

The online version contains supplementary material available at 10.1186/s12879-024-09459-x.

## Introduction

The World Health Organization (WHO) lists antimicrobial resistance (AMR) as one of the top ten main threats to global health [1]. Each year, AMR contributes to 700 000 deaths worldwide; and if nothing is done, the death toll is estimated to reach 10 million by the year 2050 [1, 2]. Carbapenem-resistant Enterobacterales (CREs) are among WHO priority AMR pathogens that require urgent attention, research, and the development of new antimicrobials [2]. CREs are an important cause of healthcare-associated infections, including life-threatening bloodstream infections (BSIs) and hospital-acquired pneumonia [3, 4]. In South Africa, the frequency of CRE infections among hospitalized patients continues to increase [5, 6]. Compounding the problem is the increasing resistance to available antibiotics, which threatens the efficacy of already limited treatment options and increases the risk of mortality [7].

While newer antibiotics such as plazomicin, ceftazidime-avibactam (CA), meropenem-vaborbactam, cefiderocol, and eravacycline have been developed, these are not widely available in resource-limited settings [8–10]. Regulatory impediments and the high cost of new antimicrobial agents contribute to the lack of availability and usage of new antimicrobials in low- and middle-income countries [11, 12]. Therapeutic options are therefore limited to a few mainstay drugs such as polymyxins or colistin [13–15]. In South Africa, the available treatment options include colistin, tigecycline, carbapenem, and β-lactamase inhibitor combinations (BLICs) such as ceftolozane-tazobactam, and ceftazidime-avibactam, although colistin is the most widely available antimicrobial, particularly in public-sector facilities [16–18]. Nonetheless, treatment practices for Gram-negative infections and CREs likely vary owing to conflicting available evidence on optimal treatment and lack of standardised treatment guidelines [16, 18–20].

Although colistin has been associated with reduced mortality and suppression of emerging resistance, nephrotoxicity and neurotoxicity in critically ill patients make this drug less desirable and necessitates consideration of alternative drugs with fewer adverse effects [21]. However, the conflicting evidence on whether regimens without colistin are inferior to colistin-based therapy for CRE infections makes it difficult to recommend alternatives despite the challenges with associated with colistin [13, 22]. In a meta-analysis of observational studies of patients with carbapenemase-producing Enterobacterales bloodstream infections, those treated with combination therapy had a lower mortality risk than those treated with monotherapy (risk ratio 0.61, 95% confidence interval [CI], 0.45-0.85) [23]. However, it remains unclear if this mortality benefit was specific to colistin because multiple combination therapies were studied, including colistin-based combinations, carbapenem-based combinations, tigecycline-based combinations, and others [23]. In South Africa, small single-center cross-sectional studies of patients prescribed colistin have shown high in-hospital mortality (>40%) in patients treated with either colistin mono- or combination therapy [16, 18]. Nonetheless, South African studies utilizing analytical methods to evaluate the influence of colistin therapy on mortality have not been documented.

This study aimed to describe antimicrobials prescribed to patients with CRE bacteremia in South African public-sector hospitals and assess the impact of treatment regimens on mortality. We hypothesised that patients treated with colistin-sparing therapy would have higher odds of in-hospital mortality compared to those treated with colistin-based therapy.

## Methods

### CRE surveillance

We analysed secondary data collected in the cross-sectional GERMS-SA CRE surveillance study conducted from January 2015 to December 2020. Detailed methods for the GERMS-SA CRE surveillance have been described previously [24]. Briefly, Surveillance for CRE bloodstream infections was conducted at enhanced sentinel sites (ESS) across four provinces, including secondary and tertiary public sector hospitals in KwaZulu-Natal, Gauteng, Free State, and Western Cape provinces. Patients admitted to ESS with Enterobacterales cultured from a blood culture specimen and phenotypically resistant to one or more carbapenems (ertapenem, imipenem, meropenem, and/or doripenem) were included in the surveillance program, no other specimen types were included. Clinical data of the case-patients were collected through medical record review and/or patient interviews by surveillance officers (SOs) using standard case report forms following an informed consent. The variables collected by SOs on the CRF included, but were not limited to age, sex, ward, pre-existing medical condition, and medical devices. Data were collected at a single time point and patients were not followed-up. Project coordinators conducted quality checks for each CRF and if any queries arose, they liaised with the relevant SO for correction and completion. Only CRFs that passed the quality control process were utilized in the final analysis. Diagnostic laboratories submitted CRE isolates to the National Institute for Communicable Diseases (NICD) reference laboratory. Bacterial identification of submitted isolates was done using the Microflex matrix-assisted laser desorption/ionization time-of-flight mass spectrometry (MALDI-TOF) (Bruker Daltonik GmbH, Germany). Antimicrobial susceptibility testing was performed using the MicroScan Walkaway system (Siemens Healthcare Diagnostics Inc., USA) and the Sensititre instrument (Trek Diagnostic Systems Ltd, UK) with the FRCOL panel (Separation Scientific SA (Pty) Ltd, SA) was used for confirmation of colistin resistance. Minimum inhibitory concentrations were interpreted according to the 2020 Clinical & Laboratory Standards Institute guidelines. Carbapenemase genes were detected using a multiplex real-time polymerase chain reaction (PCR) assay (LightCycler 480 II; Roche Diagnostics Corp., USA).

### Key definitions

A colistin-based regimen included colistin alone or colistin plus one or more other antimicrobials indicated for the treatment of Gram-negative infections (carbapenems, tetracycline, and/or aminoglycosides). Colistin-sparing therapy included a combination of or monotherapy with carbapenems, tetracycline, and/or aminoglycosides.

### Study inclusion and exclusion criteria

For this study, we included all case-patients with confirmed carbapenem resistance by the reference laboratory. If an already included case-patient had a CRE isolate identified subsequently at least 21 days after the initial positive test, they were included as additional cases. We excluded case-patients with carbapenem-susceptible isolates or missing data for treatment and outcome.

### Data analysis

Data analysis was performed using Stata version 15 (StataCorp LP, College Station, Texas, USA). Demographic and clinical characteristics of CRE bacteremia case-patients, such as age, sex, province, CRE genes, risk factors, and patient outcomes, were summarized using descriptive statistics. Categorical and continuous variables of patients treated with different regimens were compared using the Chi-square test and the Mann-Whitney-Wilcoxon test, respectively. To assess the association between mortality and colistin-based combination therapy versus colistin-sparing therapy, first, a bivariable logistic regression analysis was performed while controlling for a third confounding variable. Potential confounder variables of interest included age, sex, intensive care unit (ICU) admission, pre-existing medical condition, human immunodeficiency virus (HIV) status, and mental status at diagnosis. The change in the main effect estimate (odds ratio [OR]) when adjusting for each third variable was assessed. Variables that resulted in a ≥10% change in the main effect during bivariable analysis were included in the multivariable model. *A priori* confounders (age, sex, and ICU admission) were also included in the final model regardless of their impact on the main effect. In the final model, missing data were accounted for using multiple imputations on binary predictor variables (mental status, ICU admission, and underlying medical conditions) using the method of chained equations. Fifty sets of multiply imputed data were generated. Potential differences in the level of care were accounted for by adjusting for clustering by hospital. Adjusted odds ratios (aORs) from the final model were reported with the corresponding 95% confidence intervals (CIs) and *p*-values.

### Ethical considerations

The GERMS-SA enhanced CRE surveillance primary study was approved by the Human Research Ethics Committee (Medical) of the University of the Witwatersrand (No: M10464), and the surveillance officers obtained informed consent from participants during the data collection process. This nested study received approval from the Faculty of Health Sciences Research Ethics Committee (FHSREC) at the University of Pretoria (UP) (116/2021). Data for this study were de-identified, and unique identifiers were used for analysis.

## Results

### Overview of cases of CRE bacteremia

**Fig. 1:**
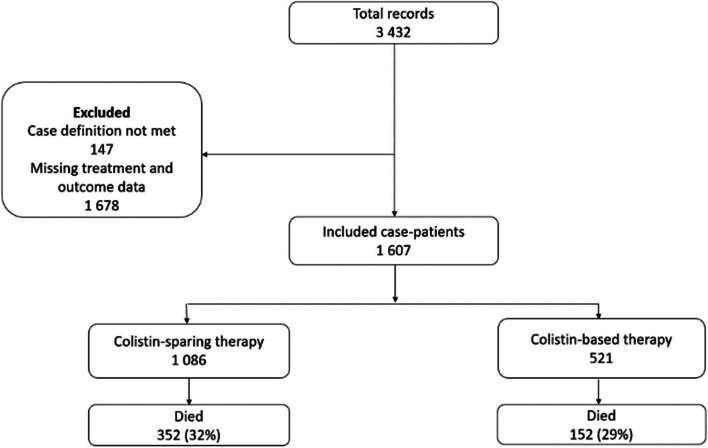
Treatment regimens of case-patients with carbapenem-resistant Enterobacterles bacteremia at GERM-SA sentinel sites, South Africa, January 2015 – December 2020

During the five-year study period, there were 3 432 surveillance records and we included 1 607 (46.8%) CRE case-patients after applying exclusions (Fig. 1).

**Fig. 2:**
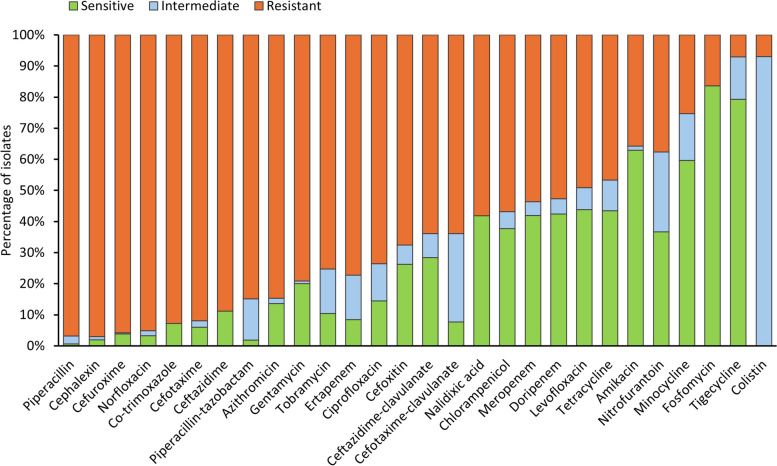
Antimicrobial susceptibility of carbapenem-resistant Enterobacterales isolates from patients with bacteremia at GERM-SA enhanced sentinel site, South Africa, January 2015 – December 2020

The median age was 29 years (interquartile range [IQR], 0-50 years) 53% (857/1 086) of the cases were male (Table 1). Of the case-patients aged <10 years (*n*=570), neonates (age 0-27 days) accounted for 57% (327/570). The majority of the cases were from sites in Gauteng Province (64% [1 034/1 607]) followed by sites in KwaZulu-Natal Province (21% [327/1 607]). Of the 1 546 organisms identified, the most common causative pathogen was *Klebsiella pneumoniae* (82%, *n*=1 247), followed by *Enterobacter clocae* (5%, *n*=82) and *Serratia marcescens* (5%, *n*=81) (Appendix figure 1). The highest antimicrobial resistance was observed for piperacillin (97%, 882/909), and cephalexin (97%, 257/264). Isolates exhibited the lowest resistance to colistin (7%, 17/253), tigecycline (7%, 60/910), and fosfomycin (16%, 142/910) (Fig. 2). A majority of the isolates harbored bla_OXA-48 & variants_ (61%, *n*=551), followed by bla_NDM_ (37%, *n*=333) and bla_VIM_ (2%, *n*=22). Most case-patients had an underlying condition (54% [784/1 521]) and 24% (285/1 202) were HIV positive. Just over a quarter of case-patients were admitted to an ICU (31%, 435/ 1 418) at the time of positive specimen collection and nearly half (45% [646/1 435]) were exposed to broadspectrum antibiotics in the past six months. Most case-patients received colistin-sparing regimens (68% [*n*=1 086]) and 33% (*n*=521) received a colistin-based regimen. Compared to patients on colistin-sparing regimen, the highest proportion of patients on colistin-based therapy were aged <10 years (44% [232/521] versus 31% [338/1 086], *p*<0.001) and were admitted to an ICU (29% [129/442] versus 31% [306/976], *p*=0.40). The overall in-hospital mortality was 31% (504/1 607) and was marginally higher among patients treated with colistin-sparing therapy compared to patients treated with colistin-based therapy (32% [352/1 086] vs 29% [152/521], *p*=0.18) however, the difference did not reach statistical significance. Patients on colistin-based therapy had longer time to death from specimen collection compared to patients on colistin-sparing regimen (median of 13 days versus 8 days, *p*<0.001) (Table 1).

**Table 1:** Demographic and clinical characteristics of cases of carbapenem-resistant Enterobacterales bacteremia at GERM-SA sentinel sites by treatment regimen, South Africa, January 2015 – December 2020

**Characteristics**	**All** ***N*=1 607**	**Colistin-sparing therapy** ***n*=1 086**	**Colistin-based therapy** ***n*=521**	***p*-value**
	**n (%)**			
**Age groups in years**	1 086	1 086	521	
<10	570 (36)	338 (31)	232 (44)	<0.001
10–20	96 (6)	52 (5)	44 (8)	
21–30	166 (10)	111 (10)	56 (11)	
31–40	223 (14)	159 (15)	64 (12)	
41–50	160 (10)	120 (11)	40 (8)	
>50	392 (24)	306 (28)	86 (17)	
**Sex**	1 086	1 086	521	
Male (vs. female)	857 (53)	564 (52)	293 (56)	0.11
**Province**^**a**^	1 607	1 086	521	
Gauteng	1034 (64)	702 (65)	332 (64)	<0.001
KwaZulu Natal	327 (21)	222 (20)	105 (20)	
Western Cape	223 (14)	147 (14)	76 (15)	
Free State	23 (1)	15 (1)	8 (2)	
**CPE**^**b**^** genes**	909	630	279	0.009
bla_OXA-48-like_	551 (61%)	404 (64)	147 (53)	
bla_NDM_	333 (37%)	209 (33)	124 (44)	
bla_VIM_	22 (2%)	16 (3)	6 (2)	
bla_GES_	2 (0%)	1 (0)	1 (0)	
bla_KPC_	1 (0%)	0 (0)	1 (0)	
**Antibiotic exposure (prior 6 months)**	1 435	962	473	0.064
Yes (vs. no)	646 (45)	417 (43)	229 (49)	
**Any underlying condition**^**c**^	1 521	1 033	488	0.059
Yes (vs no)	784 (54)	573 (55)	245 (50)	
**HIV status**	1 202	803	399	0.001
Positive (vs negative)	285 (24)	213 (27)	72 (18)	
**CD4 cell count**	207	154	53	0.52
≥200 cells/mm3	74 (36)	57 (37)	17 (32)	
<200 cells/mm3	133 (64)	97 (63)	36 (68)	
**Glasgow coma scale**	1 424	963	461	0.90
15	743 (52)	501 (52)	242 (53)	
<15	681 (49)	462 (48)	219 (46)	
**Medical device**	1 373	943	430	
Peripheral vascular catheters	1115 (81)	762 (81)	353(82)	0.57
Other devices^**d**^	258 (19)	181 (19)	77 (18)	
**ICU**^**e**^** admission**	1 418	976	442	0.40
Yes (vs no)	435(31)	306 (31)	129 (29)	
**Mechanically ventilated**	1 598	1 079	519	0.019
Yes	538 (34)	343 (32)	195 (38)	
**Outcome**	1 607	1 086	521	0.18
Alive	1 103 (69)	734 (68)	369 (71)	
Dead	504 (31)	352 (32)	152 (29)	
**Days to outcome**^**f**^	619	445	174	<0.001
Median days (interquartile range)	10 (3-21)	8 (2–19)	13 (6–25)	

^a^Number of sentinel sites in each province; KwaZulu-Natal (*n*=5), Gauteng (*n*=5), Free State (*n*=1) and Western Cape (*n*=2)

^b^Carbapenemase-producing Enterobacterales

^c^Underlying conditions included malignancy, cardiovascular disease, renal failure, and diabetes mellitus

^d^Other medical devices included central venous lines, urinary catheters, drainage ports, and/or intra-arterial lines

^e^Intensive care unit

^f^From specimen collection to death

### Antibiotics prescribed

Overall, meropenem was the most frequently prescribed antibiotic (52%, *n*=837), followed by imipenem (37%, *n*=592), amikacin (36%, *n*=583) and colistin (32%, *n*=521) (Table 2). The median time to initiation of meropenem treatment following positive specimen collection was 0 days (IQR -4-3). The median dosage administered for colistin was 900 mg (IQR 87–4500mg) with a median of 3 days (IQR 1-5 days) to initiation of colistin treatment following positive specimen collection.

**Table 2:** Antibiotic treatment patterns among cases of carbapenem-resistant Enterobacterales bacteremia at GERM-SA enhanced sentinel site, South Africa, January 2015 – December 2020

**Antibiotic**^**a**^	**All** ***N*=1 607**	**Colistin-sparing therapy** ***n*=1 086**	**Colistin-based therapy** ***n*=522**
	n (%) and (IQR)		
**Colistin**	**521 (32)**	Not relevant	**521 (100)**
Median dosages (mg)	900 (87-4500)	Not relevant	930 (85-4500)
Median doses administered	5 (2-8)	Not relevant	5 (2-8)
Days to treatment^b^	3 (1-5)	Not relevant	3 (1-5)
**Ertapenem**	**257 (16)**	**211 (19)**	**46 (9)**
Median dosage (mg)	1000 (500-1000)	1000 (500-1000)	1000 (500-1000)
Median doses administered	4 (2-7)	4 (2-7)	3 (2-7)
Days to treatment	0 (-7-1)	0 (-8-1)	0 (-7-2)
**Imipenem**	**592 (37)**	**426 (39)**	**166 (32)**
Median dosage (mg)	500 (500-1000)	775 (500-1000)	500 (300-1000)
Median doses administered	6 (3-9)	6 (3-9)	6 (3-10)
Days to treatment	1 (-2-3)	1 (-2-3)	2 (-1-4)
**Meropenem**	**837 (52)**	**503 (46)**	**335 (64)**
Median dosages (mg)	500 (94-1000)	750 (100-1000)	500 (90-1000)
Median doses administered	7 (3-11)	6 (3-10)	7 (4-12)
Days to treatment	0 (-4-3)	0 (-5-3)	1 (-2-3)
**Gentamicin**	**187 (11)**	**150 (14)**	**37 (7)**
Median dosages (mg)	320 (62-5400)	400 (60-5500)	240 (65-5000)
Median doses administered	4 (2-6)	4 (2-6)	3 (3-6)
Days to treatment	-6 (-15-0)	-6 (-15-0)	-7 (-13-0)
**Amikacin**	**583 (36)**	**442 (41)**	**141 (27)**
Median dosages (mg)	500 (50-1000)	500 (50-1000)	230 (35-1000)
Median doses administered	5 (2-8)	5 (2-7)	4 (2-7)
Days to treatment	0 (-4-3)	1 (-2-3)	0 (-4-2)
**Piperacillin/Tazobactam**	**478 (30)**	**366 (34)**	**112 (22)**
Median dosages (mg)	2250 (320-4500)	2250 (440-4500)	1350 (220-4500)
Median doses administered	5 (3-8)	5 (2-8)	5 (3-7)
Days to treatment	-3 (-8-1)	-2 (-8-1)	-4 (-12-0)
**Amoxicillin-clavulanate**	**247 (15)**	**200 (18)**	**47 (9)**
Median dosages	1200 (625-1200)	1200 (1000-1200)	1200 (600-1200)
Median doses administered	5 (3-7)	5 (3-7)	5 (3-8)
Days to treatment	-7 (-16-(-2_)	-7 (-16-(-2))	-9 (-17- (-10))
**Azithromycin**	**66 (4)**	**45 (4)**	**21(4)**
Median dosages	500 (250-500)	500 (500-500)	500 (35-500)
Median doses administered	5 (3-7)	5 (3-7)	5 (3-6)
Days to treatment	-3 (-14-2)	-4 (-11-1)	-1 (-21-4)

^a^Data for some antibiotics were not shown due to low numbers: tigecycline, *n*=10; piperacillin, *n*=10; doxycycline, *n*=7; tobramycin, *n*=2

^b^Median days to antibiotic initiation relative to the positive specimen collection

### The association of treatment with in-hospital mortality

In our unadjusted model of the association between treatment categories and in-hospital mortality, colistin-based therapy (OR=0.99; 95% CI: 0.74–1.34, *p*-value=0.191) had lower odds of mortality compared to colistin-sparing therapy, however the association did not reach statistical significance. After adjusting for age, sex, mental status at diagnosis, underlying conditions, and ICU admission, the odds of in-hospital mortality among those treated with colistin-based therapy compared to colistin-sparing therapy were 1.02 (95% CI, 0.78–1.33, *p*=0.873) (Table 3).

**Table 3:** Multivariable logistic regression model for the association between in-hospital mortality and treatment regimen among patients with carbapenem-resistant Enterobacterales bacteremia at GERMS-SA enhanced sentinel site, 2015-2020

**Characteristics**	**Case fatality ratio**	**Univariate analysis**		**Multivariate analysis**	
		**OR**^**a**^** (95% CI**^**b**^**)**	***p*-value**	**aOR**^**c**^** (95% CI)**	***p*-value**
Colistin-sparing therapy^d^	352/1086 (32)	Reference			
Colistin-based therapy^e^	152/521 (29)	0.99 (0.74-1.34)	0.191	1.02 (0.78-1.33)	0.873
**Age groups**					
<10	115/570 (20)	Reference			
10-20	28/96 (29)	1.62 (1.00-2.64)	0.000		
21-30	49/167 (29)	1.64 (1.11-2.42)			
31-40	76/223 (34)	2.04 (1.44-2.88)			
41-50	56/160 (35)	2.13 (1.45-3.12)			
>50	180/392 (46)	3.35 (2.52-4.46)			
**Sex**					
Male	251/857 (29)	Reference	0.056		
Female	253/751 (34)	1.23 (0.99-1.51)			
**Mental status (GCS**^**f**^**)**					
15	127/743 (17)	Reference			
<15	308/682 (45)	3.99 (3.13-5.09)	0.000		
**Comorbidities**					
No	166/703 (23)	Reference			
Yes	312/818 (38)	1.99 (1.59-2.49)	0.000		
**ICU admission**					
No	277/984 (28)	1.81 (1.43-2.30)	0.000		
Yes	181/435 (42)				

^a^Odds ratio

^b^Confidence interval

^c^Adjusted odds ratio

^d^Colistin-sparing therapy included carbapenems, tetracycline and/or aminoglycosides

^e^Colistin-based therapy included colistin plus a carbapenem(s) or colistin plus an aminoglycosides (s)

^f^Glasgow coma scale

## Discussion

In this study, patients with CRE bacteremia were treated with various antibiotics, the majority with colistin-sparing therapy, with meropenem being the most frequently prescribed drug. Colistin was frequently given to younger patients and patients on mechanical ventilation. In contrast to other antibiotics that were given on average prior to specimen collection, colistin was given a few days following the specimen collection. The in-hospital mortality rate was marginally lower among those treated with colistin-based therapy compared to those with colistin-sparing therapy; however, there were equal odds of in-hospital mortality for patients on both therapy regimens.

The colistin-sparing therapy, mainly containing meropenem, was the most commonly prescribed regimen in our study. This was contrary to the case–control study conducted in Istanbul, Turkey where most patients were treated with colistin-based combinations (12% versus 7% for non-colistin therapy) [25]. The high proportion of patients given meropenem indicates its frequent use as empiric therapy due to the high prevalence of extended-spectrum β-lactamase producing Enterobacterales among patients with bacteremia in South African hospitals [26, 27]. In addition, treatment with other drugs before or on the specimen collection was likely empiric, while colistin was likely based on laboratory confirmation of CRE infection a few days following specimen collection. It was surprising that colistin was not prescribed for a majority of the patients, even following laboratory confirmation of CRE infection. The shorter time to mortality observed in patients who received colistin-sparing regimens may explain why colistin wasn't administered in this group, potentially due to patients having already demised before consideration of colistin inclusion. Nonetheless, in our setting, the choice of antibiotics other than colistin may be influenced by the availability; colistin is a section 21 drug that can only be obtained through motivation to the Medicines Control Council, a process that may deter clinicians from prescribing the drug [19]. Indeed, a majority of patients who received colistin-based therapy tended to fall within younger age groups or were mechanically ventilated, which may signal critical illness or perceived high risk of death prompting inclusion of colistin. In addition, the variation in the choice of antibiotics and non-inclusion of colistin could be the due to lack of clear non-explicit standard guidelines on the treatment CRE infections.

We found a lower mortality for patients receiving colistin-based therapy compared to those on colistin-sparing therapy, and our adjusted analysis showed a modest 10% decrease in the odds of mortality. However, the confidence intervals showed that equal odds of mortality in the treatment groups could not be excluded. The lack of statistical significance may be attributed to missing mortality and treatment data of a large number of excluded case-patients, potentially resulting in inadequate power to detect a statistically significant difference. Nonetheless, our findings are in keeping with other observational studies showing no difference in mortality among patients treated with colistin-based and other regimens [28, 29]. Similar mortality in the treatment groups could be because colistin-sparing therapies have been shown to be effective for CRE infection [30–33]. These studies as well as our results suggests colistin-based regimens may not be better than colistin-sparing regimens in reducing the risk of death.

While colistin-sparing treatment options may not increase the risk of death, increasing resistance may limit their utility. The efficacy of combination carbapenem treatment is influenced by the minimum inhibitory concentrations (MICs) of the isolates, with the drugs being less effective at higher MICs (>8 mg/L) [34]. Alternative and effective antibiotic choices therefore remain warranted. In South Africa, ceftazidime-avibactam is the only available option among newer-generation drugs indicated for CRE infections [10]. Retrospective-multicenter studies conducted in Saudi Arabia and the United States have shown higher clinical success rate (e.g., resolution of signs and symptoms of infection, normalizing of white blood cell count and procalcitonin level, and bacterial clearance on blood culture) with the use of ceftazidime-avibactam compared to combination therapy with or without colistin [31, 35]. If newer antimicrobials are included in the arsenal of treatment options in public-sector hospitals, studies demonstrating their role in mortality and other outcomes compared to existing options would be invaluable to guide development of standardised treatment guidelines for CRE infections.

### Strengths and limitations

Our cross-sectional study used secondary data collected through the primary surveillance study, which was not optimally designed and powered for our objective. Nearly half of the CRE case-patients were excluded and these case-patients may have differed to those we included with respect to some characteristics such as HIV infection and ICU admission. The absence of data of these patients could have introduced bias into the study, potentially resulting in misleading conclusions, such as an inaccurate direction of effect or the perception of no association between treatment and mortality, or even a lack of statistical significance. However, despite differences, most variables including sex, antibiotic exposure and underlying medical conditions were similar, indicating that the cases included in our study were likely representative and that our findings are reliable. The patient's clinical condition, in the context of their treatment plan, plays a pivotal role in determining their outcome in addition to the specific combination of drugs used [19]. We analysed all-causes in-hospital mortality and could not completely account for the role of other underlying conditions, appropriateness of treatment and adverse effects. Despite the limitations, our study provided insight on treatment options used for patients with CRE bacteremia in South African hospitals, and demonstrated that patients treated with either colistin-based or colistin-sparing treatments have similar mortality risk.

## Conclusions

Colistin was not the main drug used to treat patients with CRE infection in South African public-sector hospitals and the mortality risk did not differ between patients treated with colistin-based therapy versus colistin-sparing therapy. Studies demonstrating benefits and mortality impact of older and newer alternative therapies are needed to inform treatment guidelines in low-resource settings.

### Supplementary Information

Supplementary Material 1. 

## Data Availability

All the data supporting our findings are contained within this manuscript. Datasets used in this study may be requested from the main author: Nqobile Ngoma (email: Ngomanqobile@gmail.com).

## Abbreviations

aOR: Adjusted odds ratio
BSI: Bloodstream infections
CI: Confidence interval
CHARM: Centre for Healthcare-Associated Infections, Antimicrobial Resistance and Mycoses
CPE: Carbapenemase-producing Enterobacterales
CRE: Carbapenem-resistant Enterobacterales
ESS: Enhanced sentinel sites
HSREC: Health Sciences Research Ethics Committee
HIV: Human immunodeficiency virus
ICU: Intensive care unit
IMP: Imipenemase
KPC: Klebsiella pneumoniae carbapenemases
MBL: Class B metallo-β-lactamases
NICD: National Institute for Communicable Diseases
NDM-1: New Delhi metallo- β-lactamase-1
NHLS: National Health Laboratory Services
OR: Odds ratio
PCR: Polymerase chain reaction
SAFETP: South African Field Epidemiology Training Programme
UP: University of Pretoria
VIM: Veronica integron metallo-beta-lactamases
VABP: Ventilator-associated bacterial pneumonia
