# Supplementary material for: The impact of colistin-based regimens on mortality compared to other antimicrobials in patients with carbapenem-resistant Enterobacterales bacteremia in South African hospitals: a cross-sectional study
Source: BMC Infect Dis. 2024 Jun 5;24:561. doi: 10.1186/s12879-024-09459-x (PMC11151471; doi:10.1186/s12879-024-09459-x)
Supplement: Supplementary file 1 — Supplementary Material 1. [file 12879_2024_9459_MOESM1_ESM.docx]

**Appendix**

**Table 1:** Demographic and clinical characteristics of the included and excluded cases-patients with carbapenem-resistant Enterobacterales bacteremia at GERM-SA sentinel sites by treatment regimen, South Africa, January 2015 – December 2020**.**

| **Characteristics** | | **All**  **N=3 432** | | **Included case-patients**  **n=1 607** | | **Excluded case-patients**  **n=1 825** | **p-value** | |
| --- | --- | --- | --- | --- | --- | --- | --- | --- |
|  | | **n (%)** | | | | |  | |
| **Age group in years** | | 3 432 | | 1 607 | | 1 825 | <0.001 | |
| 1-9 | | 1076 (31) | | 570(36) | | 506 (28) |  | |
| 10-20 | | 173 (5) | | 96 (6) | | 77 (4) |  | |
| 21-30 | | 347 (10) | | 166 (10) | | 181 (10) |  | |
| 31-40 | | 473 (14) | | 223 (14) | | 250 (14) |  | |
| 41-50 | | 390 (11) | | 160 (10) | | 230 (13) |  | |
| >50 | | 973 (28) | | 392 (24) | | 581 (32) |  | |
| **Sex** | | 3400 | | 1607 | | 1793 | 0.44 | |
| Male (vs. female) | | 1837 (54) | | 857 (53) | | 980 (55) |  | |
| **Province^a^** | | 3 432 | | 1 607 | | 1 825 | <0.001 | |
| Gauteng | | 2376 (69) | | 1034 (64) | | 1342 (74) |  | |
| KwaZulu Natal | | 624 (18) | | 327 (20) | | 297 (16) |  | |
| Western Cape | | 371 (11) | | 223 (14) | | 148 (8) |  | |
| Free State | | 61 (2) | | 23 (1) | | 38 (2) |  | |
| **CPE^b^ genes** | | 1 708 | | 909 | | 799 | <0.001 | |
| bla_OXA-48-like_ | | 1155 (68) | | 551 (61%) | | 604 (76) |  | |
| bla_NDM_ | | 510 (30) | | 333 (37%) | | 177 (22) |  | |
| bla_VIM_ | | 36 (2) | | 22 (2%) | | 14 (2) |  | |
| bla_GES_ | | 3 (0) | | 2 (0%) | | 1 (0) |  | |
| bla_KPC_ | | 4 (0) | | 1 (0%) | | 3 (0) |  | |
| **Antibiotic exposure (prio 6 months)** | | 1 902 | | 1 435 | | 467 | 0.094 | |
| Yes (vs. no) | | 877 (46) | | 646 (45) | | 231 (50) |  | |
| **Any underlying conditions^c^** | | 2 017 | | 1 521 | | 496 | 0.65 | |
| Yes (vs. no) | | 1079 (54) | | 818 (54) | | 261 (53) |  | |
| **HIV status** | | 1 720 | | 1 202 | | 518 | <0.001 | |
| Positive (vs. negative) | | 467 (27) | | 285 (24) | | 182 (35) |  | |
| **CD4 cell count** | 341 | | 207 | | 134 | | | 0.13 |
| ≥200 cells/mm3 | 73 (37) | | 74 (36) | | 59 (44) | | |  |
| <200 cells/mm3 | 125 (63) | | 133 (64) | | 75 (56) | | |  |
| **Glasgow coma scale** | | 1 894 | | 1 424 | | 470 | 0.68 | |
| 15 | | 981 (52) | | 743 (52) | | 240 (51) |  | |
| <15 | | 911 (48) | | 681 (48) | | 230 (49) |  | |
| **Medical device^d^** | | 2 118 | | 1 595 | | 523 | <0.001 | |
| Yes | | 2055 (97) | | 1559 (98) | | 496 (95) |  | |
| **ICU^e^ admission** | | 2 197 | | 1 418 | | 779 | <0.001 | |
| Yes | | 606 (28) | | 435 (31) | | 171 (22) |  | |
| **Mechanically ventilated** | | 2 127 | | 1 598 | | 529 | 0.13 | |
| Yes | | 697 (33) | | 538 (34) | | 159 (30) |  | |
| **Outcome** | | 2 317 | | 1 607 | | 710 | <0.001 | |
| Alive | | 1452 (63) | | 1103 (69) | | 349 (49) |  | |
| Dead | | 865 (37) | | 504 (31) | | 361 (51) |  | |
| **Days to outcome^f^** | | 1 036 | | 619 | | 417 | <0.001 | |
| Median days (Interquartile range) | | 7 (2-19) | | 9 (3-21) | | 4 (1-14) |  | |

a, Number of sentinel sites in each province; KwaZulu-Natal (n=5), Gauteng (n=5), Free State (n=1) and Western Cape (n=2).

b, Carbapenemase-producing Enterobacterales

c, Underlying conditions included malignancy, cardiovascular disease, renal failure, and diabetes mellitus

d, Medical devices included intravenous lines, central venous lines, urinary catheters, drainage ports, and/or intra-arterial lines

e, Intensive care unit

f, From specimen collection to death


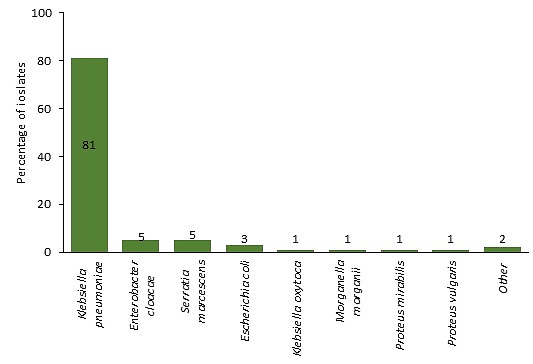


**Figure 1:** Distribution of pathogens among patients with carbapenem-resistant Enterobactereles bacteremia identified through the GERMS-SA enhanced sentinel site, South Africa, January 2015–December 2020. (*Other= Citrobacter freundii, Citrobacter koseri, Citrobacter sedlakii, Enterobacter aerogenes, Enterobacter kobei, Enterobacter asburia, Klebsiella aerogenes, Proteus penneri, Salmonella non-Typhi*)*.*
